# Itaconic acid inhibits growth of a pathogenic marine *Vibrio* strain: A metabolomics approach

**DOI:** 10.1038/s41598-019-42315-6

**Published:** 2019-04-11

**Authors:** Thao Van Nguyen, Andrea C. Alfaro, Tim Young, Saras Green, Erica Zarate, Fabrice Merien

**Affiliations:** 1 Aquaculture Biotechnology Research Group, School of Science, Faculty of Health and Environmental Sciences, Auckland University of Technology, Auckland, New Zealand; 2 Mass Spectrometry Centre, School of Biological Sciences, University of Auckland, Auckland, New Zealand; 3 AUT-Roche Diagnostics Laboratory, School of Science, Faculty of Health and Environmental Sciences, Auckland University of Technology, Auckland, New Zealand

## Abstract

The antimicrobial role of itaconic acid (ITA) has been recently discovered in mammalian cells. In our previous studies, we discovered that marine molluscs biosynthesise substantial quantities of ITA when exposed to marine pathogens, but its antimicrobial function to *Vibrio* bacteria is currently unknown. Thus, in this study, we used an untargeted gas chromatography–mass spectrometry (GC-MS) platform to identify metabolic changes of *Vibrio* sp. DO1 (*V*. *corallyliticus*/*neptunius*-like isolate) caused by ITA exposure. *Vibrio* sp. DO1 was cultured in Luria-Bertani broth supplemented with 3 mM sodium acetate and with different concentrations of ITA (0, 3 and 6 mM) for 24 h. The results showed that ITA completely inhibited *Vibrio* sp. growth at 6 mM and partially inhibited the bacterial growth at 3 mM. A principal component analysis (PCA) revealed a clear separation between metabolite profiles of *Vibrio* sp. DO1 in the 3 mM ITA treatment and the control, which were different in 25 metabolites. Among the altered metabolites, the accumulation of glyoxylic acid and other metabolites in glyoxylate cycle (cis-aconitic acid, isocitric acid and fumaric acid) together with the increase of isocitrate lyase (ICL) activity in the 3 mM ITA treatment compared to the control suggest that ITA inhibited *Vibrio* sp. growth via disruption of central carbon metabolism.

## Introduction

Itaconic acid (ITA), or 2-methylenesuccinic acid, is an unsaturated dicarboxylic acid that is a well-known precursor for polymer synthesis in the industrial production of polymers. In addition, ITA is known to have antimicrobial function which was first described in Gram-negative bacterium *Vogesella indigofera*^1^. The inhibitory effect of ITA was subsequently reported in other bacteria, including *Pseudomonas indigofera*^1,2^, *Yersinia pestis*^3^, *Mycobacterium tuberculosis* and *Salmonella enterica*^4^.

Recently, ITA was surprisingly discovered in mammalian immune cells. Shin, *et al*.^5^ reported the presence of ITA in lung tissue of mice infected with *M*. *tuberculosis*, and it was hypothesized that ITA was originated from the bacteria in this association. However, Sugimoto, *et al*.^6^ subsequently detected ITA in mouse macrophage-like cell lines stimulated with lipopolysaccharide (LPS), which demonstrated an intracellular source. The biological function of ITA as a novel mammalian metabolite was then highlighted by Strelko, *et al*.^7^ who suggested its roles in macrophage-based immune functions after observing increased ITA production and secretion in mouse peritoneal macrophages activated by LPS and IFN-γ. Similarly, the highly increased levels of ITA in human primary macrophages under LPS-induced inflammatory conditions^4^. Taken together, these findings indicate the role of ITA as mammalian antimicrobial metabolite.

In addition to mammalian macrophages, increased biosynthesis of ITA was recently reported in marine bivalves during pathogen challenges^8–10^. The first detection of ITA in bivalves was reported by Young, *et al*.^10^ in Pacific oyster larvae challenged with a marine herpesvirus (OsHV-1 µVar). Accumulation of ITA was then detected in mussel haemolymph following an *in vivo* experimental challenge with a pathogenic strain of *Vibrio* sp.^8,9,11^. Interestingly, ITA increased during initial stages of infection in mussels then decreased in those individuals which survived and recovered from the infection after a week^9^. These results suggest that ITA could be a potential biomarker for pathogen infections and health status of molluscan hosts^9^. Taken together, these studies demonstrate that marine bivalves have the capacity to synthesize ITA, with potential immune functions during pathogen infections. However, it is currently unknown whether ITA can inhibit growth of specific pathogenic marine bacteria, as it does in some terrestrial strains.

*Vibrio* is a genus of Gram-negative bacteria, possessing a number of pathogenic strains that associated with infectious diseases in marine bivalves^12^. To test the potential inhibitory role of ITA on growth of a virulent *Vibrio* strain, we cultured *Vibrio* sp. DO1 in different concentrations of ITA. This *Vibrio* strain (*Vibrio coralliilyticus*/*neptunius*-like isolate, Genbank: EU358784) was isolated from *Perna canaliculus* larvae^13^ have been showed to be pathogenic to both *P*. *canaliculus* larvae^14^ and adults^8,9,11^. GC-MS-based metabolomics was performed to compare metabolite profiles of the *Vibrio* sp. cultures and evaluate mechanistic effects of ITA on bacterial metabolism.

## Results

### Effect of ITA on the growth of *Vibrio* sp. DO1

**Figure 1:**
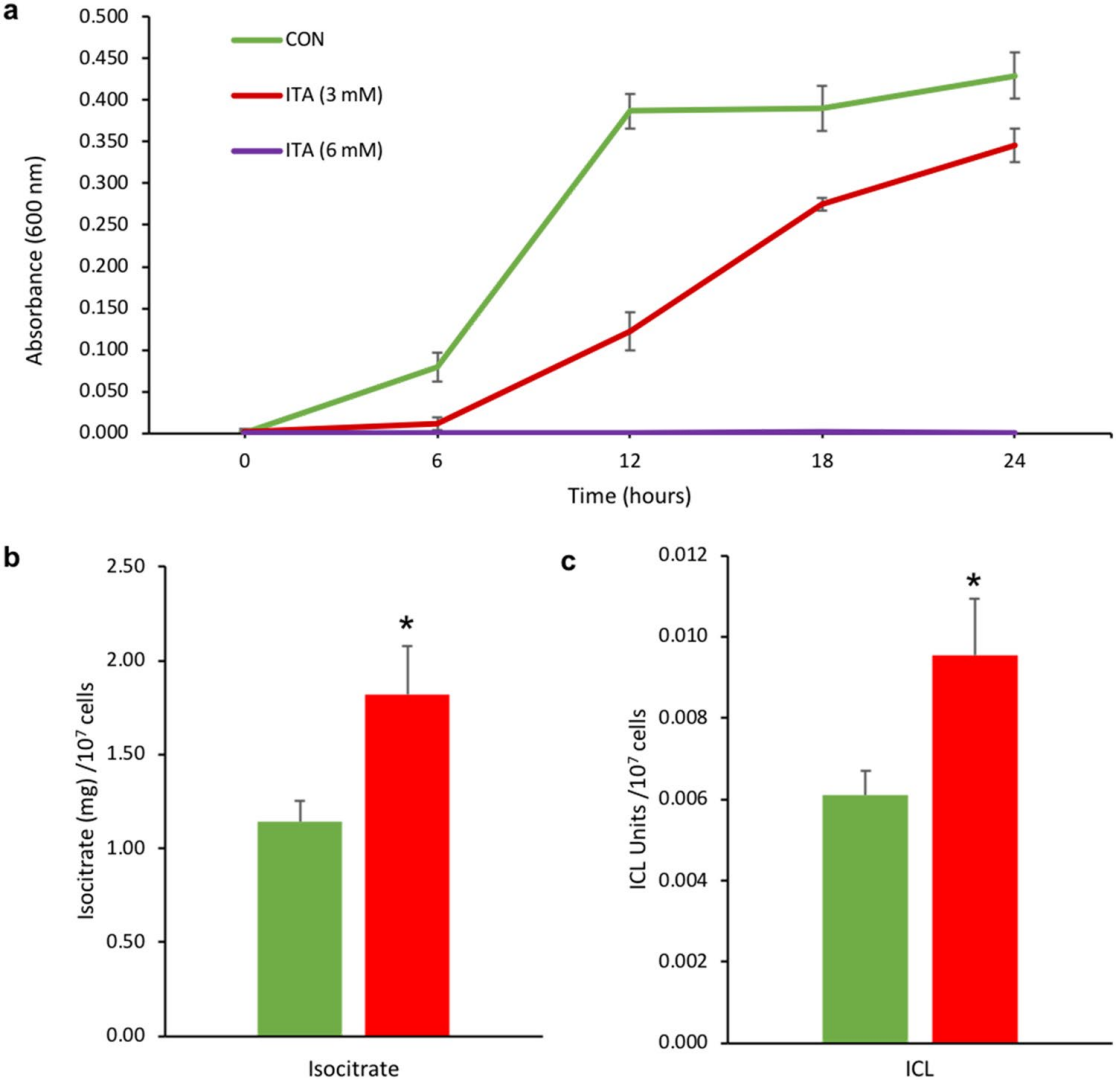
Effects of ITA on growth of *Vibrio* sp. and isocitrate and isocitrate lyase (ICL). (**a**) The absorbance (600 nm) of *Vibrio* sp. cultured in different ITA concentrations (0, 3 and 6 mM) supplemented with sodium acetate over 24 h. (**b**) Level of isocitrate in the 3 mM ITA treatment and the control at 24 hpi. (**c**) Activity of ICL in the 3 mM ITA treatment and the control at 24 hpi. Data are presented as mean ± S.D. (n = 6). Significant differences relative to the control are marked with an asterisk (*) (*t*-test, *p* < 0.05).

To test the antimicrobial effect of ITA on bacterial growth, we cultured *Vibrio* sp. in a Luria-Bertani (LB) broth with different concentrations of ITA (0, 3 and 6 mM). Luria-Bertani broth was chosen since growth in this medium is carbon limited^15^. Growth of *Vibrio* sp. was measured via spectroscopy every 6 h for 24 h (Fig. 1a). The results show that the growth of *Vibrio* sp. was completely inhibited in the 6 mM ITA treatment. Growth in the 3 mM ITA treatment was significantly slower than the growth in the control at all recorded times (*p* < 0.05). Furthermore, to test whether ITA has the capacity to inhibit isocitrate lyase (ICL) of the glyoxylate shunt, we measured the concentrations of isocitrate and activity of ICL at 24-hour post-incubation (hpi). The results show the significantly higher levels of isocitrate (t_10_ = −5.878, *p* < 0.001) and ICL activity (t_8_ = −12.52, *p* < 0.001) in the ITA treatment compared to the control (Fig. 1b,c).

### Effect of ITA on metabolite profiles of *Vibrio* sp. DO1

**Figure 2:**
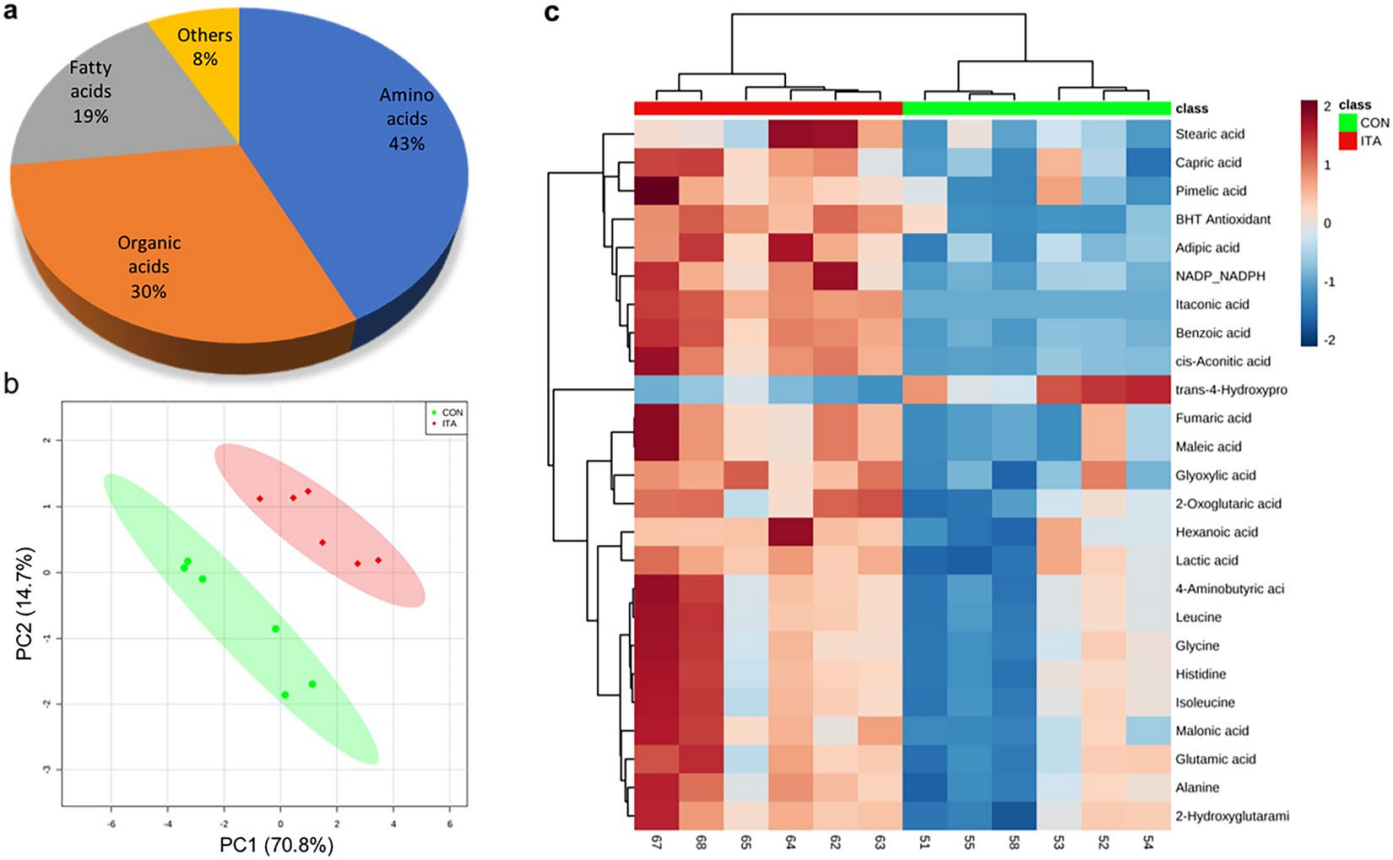
Effects of ITA on metabolite profiles of *Vibrio* sp. in the 3 mM ITA treatment and the control. (**a**) Classification of the metabolites. (**b**) PLS-DA score plot. (**c**) Heatmap of 25 metabolites identified as significantly different between the ITA treatment and the control by t-test (*p* < 0.05). CON, control treatment (no ITA); ITA, 3 mM ITA treatment.

Untargeted GC-MS-based metabolomics was performed to compare metabolite differences between the 3 mM ITA treatment and the control. A total of 565 features were detected by GC-MS in *Vibrio* sp. samples and 63 metabolites were successfully annotated using an in-house library. The majority of these metabolites were amino acids (43%), followed by organic acids (30%), fatty acids (19%) and others (8%) (Fig. 2a).

Principal component analysis (PCA) was used to identify natural groupings of all bacterial samples based on the underlying structure of the metabolite data. A PCA score plot shows a clear separation between the ITA treatment and the control (Fig. 2b). Partial least-squares discriminant analysis (PLS-DA) was revealed a very robust model for discrimination between sample classes with an accuracy of 1.0, a multiple correlation coefficient (R^2^) of 0.99 and a cross-validated predictive ability (Q^2^) of 0.90. *T*-tests identified 25 metabolites that were significantly altered by ITA treatment, compared to control cultures (Table S1). A heatmap was generated to visualise the relative abundance of these metabolites in each group (Fig. 2c). Overall, clear differences were observed between the treatment and the control, where most of the metabolites in the ITA treatment were elevated with exception of trans-4-hydroxyproline.

### Effect of ITA on the glyoxylate shunt

**Figure 3:**
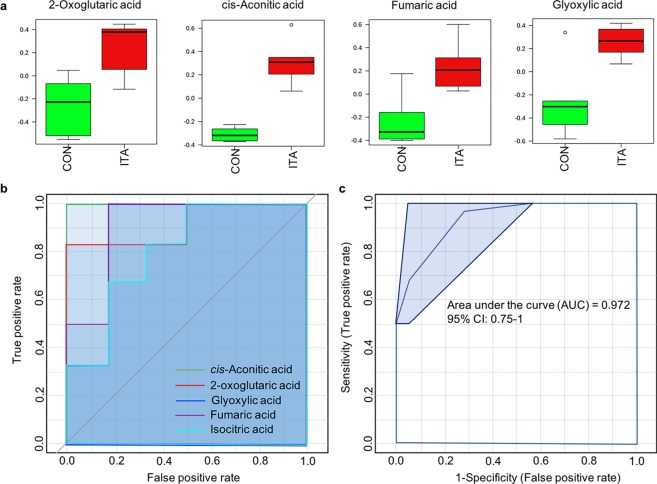
Effects of ITA on glyoxylate shunt of *Vibrio* sp. cultured in LB media with and without ITA. (**a**) Altered metabolites of glyoxylate shunt identified by *t*-test (*p* < 0.05). Box plots show relative abundances of metabolites after normalization (**b**) Univariate ROC curve analysis of significantly altered metabolites (ITA/Non ITA) of the glyoxylate shunt. (**c**) Multivariate ROC curve-based model evaluation of all altered metabolites in the glyoxylate shunt identified by ROC curve analysis.

Eight metabolites related to the glyoxylate shunt and TCA cycle were identified in metabolite profiles of *Vibrio* sp., including citric acid, cis-aconitic acid, isocitric acid, succinic acid, fumaric acid, malic acid, glyoxylic acid, pyruvic acid, 2-oxoglutaric acid and 2-phosphoenolpyruvic acid. The addition of ITA led to alterations of 4 metabolites which were elevated in the ITA treatment, including cis-aconitic acid, fumaric acid, glyoxylic acid and 2-oxoglutaric acid (Fig. 3a).

Classical univariate ROC curve analyses were performed to assess the biomarker specificity and sensitivity of these significantly altered metabolites based on the area under the ROC curve (AUC). The results revealed that all these metabolites had AUC higher than 0.8, and cis-aconitic acid had AUC equal to 1 (Fig. 3b). In addition, ROC curve analyses identified isocitric acid as significantly different between the treatment and the control with *t*-test p-value < 0.05 and AUC = 0.81. The multivariate ROC curve-based model evaluation, which combined potential biomarkers, showed a very high AUC of 0.972.

### Pathway analysis

Pathway analysis was used to identify potentially altered metabolic pathways induced by ITA. This analysis identified 28 pathways with *p* < 0.05, which contained at least two identified metabolites (Table 1). Among these, 15 pathways with impacts >0.1 were considered as pathways of interest relating to ITA effects (Table 1), while those with an impact <0.01 may have slight effects. These pathways are involved in carbohydrate metabolism (e.g., TCA cycle, pyruvate metabolism), energy metabolism (e.g., nitrogen metabolism, sulfur metabolism), amino acid metabolism (e.g., valine, leucine and isoleucine biosynthesis; arginine and proline metabolism), lipid metabolism (e.g., biosynthesis of unsaturated fatty acids), oxidative stress (e.g., glutathione metabolism), metabolism of cofactors and vitamins (e.g., pantothenate and CoA biosynthesis), among others.

**Table 1:** List of metabolic pathways in *Vibrio* sp. that were significantly affected by ITA exposure.

Pathways	Hits/Total compounds	Raw p	FDR	Impact
Alanine, aspartate and glutamate metabolism	6/18	<0.001	<0.001	0.479
Glycine, serine and threonine metabolism	6/32	0.001	0.001	0.473
Pyruvate metabolism	3/26	0.005	0.007	0.438
Glutathione metabolism	5/21	<0.001	<0.001	0.381
Citrate cycle (TCA cycle)	8/20	<0.001	<0.001	0.301
Butanoate metabolism	4/18	<0.001	0.001	0.255
Cysteine and methionine metabolism	6/34	0.001	0.001	0.230
Arginine and proline metabolism	8/41	<0.001	0.001	0.215
Glycolysis or Gluconeogenesis	2/29	0.192	0.192	0.168
Methane metabolism	2/11	<0.001	<0.001	0.167
Aminoacyl-tRNA biosynthesis	18/66	<0.001	<0.001	0.130
Glyoxylate and dicarboxylate metabolism	5/29	<0.001	<0.001	0.102
Nicotinate and nicotinamide metabolism	2/13	0.001	0.001	0.089
Sulfur metabolism	2/13	0.001	0.001	0.069
Valine, leucine and isoleucine biosynthesis	5/26	<0.001	<0.001	0.036
C5-Branched dibasic acid metabolism	2/6	<0.001	<0.001	0.000
Benzoate degradation via CoA ligation	3/10	<0.001	<0.001	0.000
Valine, leucine and isoleucine degradation	3/23	<0.001	<0.001	0.000
beta-Alanine metabolism	2/16	<0.001	0.001	0.000
Nitrogen metabolism	5/18	<0.001	0.001	0.000
Phenylalanine, tyrosine and tryptophan biosynthesis	4/23	<0.001	0.001	0.000
Thiamine metabolism	2/19	<0.001	0.001	0.000
Lysine biosynthesis	2/13	<0.001	0.001	0.000
Cyanoamino acid metabolism	4/8	0.001	0.001	0.000
Phenylalanine metabolism	4/23	0.001	0.001	0.000
Pantothenate and CoA biosynthesis	4/23	0.001	0.001	0.000
Tyrosine metabolism	2/10	0.002	0.003	0.000
Biosynthesis of unsaturated fatty acids	3/6	0.011	0.015	0.000

## Discussion

**Figure 4:**
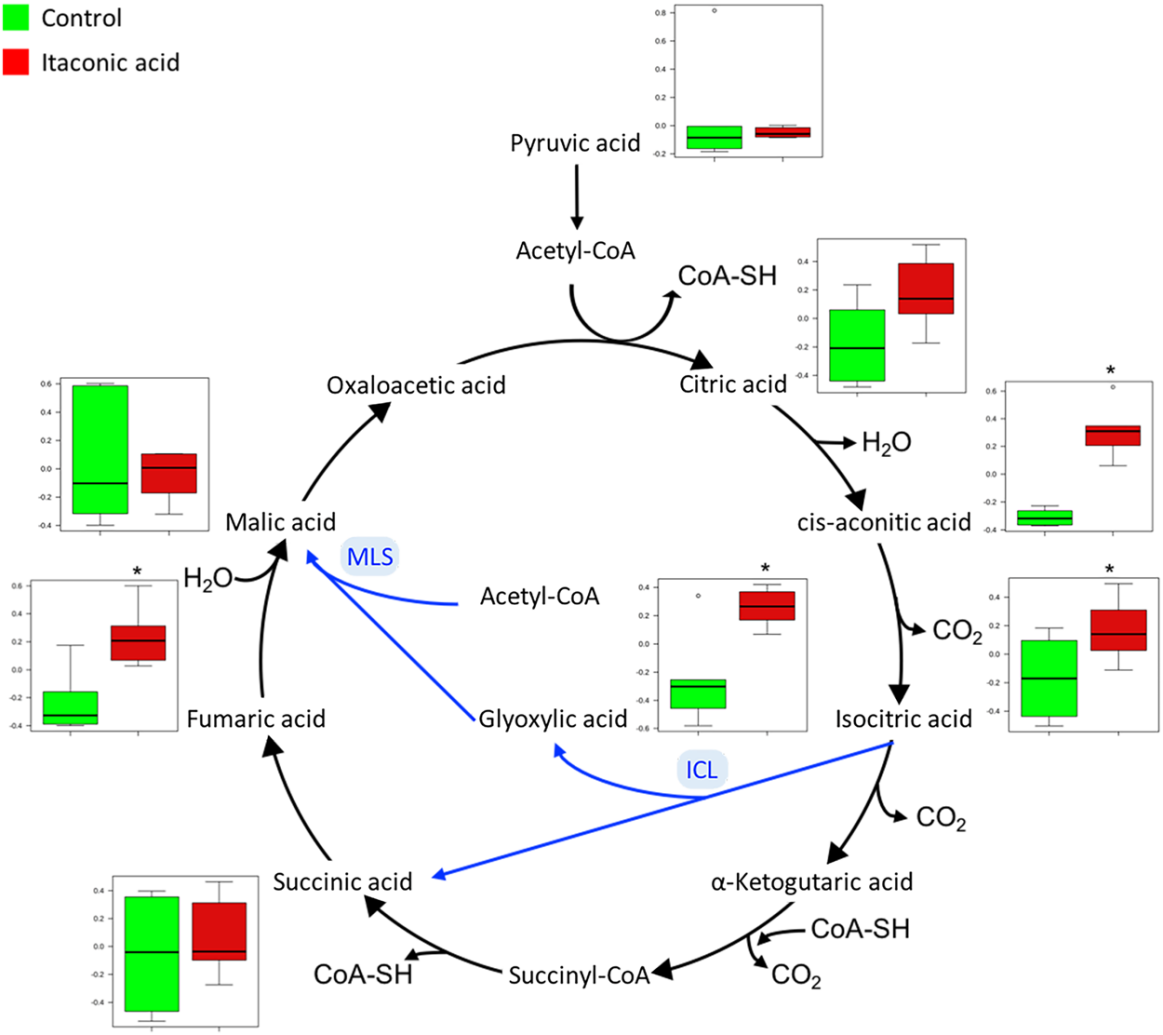
The general scheme for tricarboxylic acid (TCA) cycle (black arrows) and glyoxylate cycle (blue arrows). Box plots show relative abundances of metabolites after normalization and significant differences relative to the control are marked with an asterisk (*) (*t*-test, *p* < 0.05).

The glyoxylate cycle is an anabolic variation of the TCA cycle used during carbon limitation in plants, bacteria, protist and fungi^16^. This mechanism differs from the TCA cycle in which the two decarboxylation reactions (isocitrate → α-ketoglutarate → succinyl-CoA) are bypassed via the glyoxylate shunt pathway (Fig. 4). In this pathway, isocitric acid is converted into glyoxylic acid and succinic acid by isocitrate lyase (ICL). Glyoxylic acid is further combined with acetyl-CoA to form malic acid by malate synthase (MLS)^17,18^. When initiated during glycolytic sugar (C5-6) starvation, bypassing the CO_2_-producing steps of the TCA cycle helps the glyoxylate shunt preserve carbon atoms of acetyl-CoA for gluconeogenesis, which is critical for biomass production^19^. The glyoxylate cycle thus allows microorganisms to utilize carbon compounds other than glucose, such as acetate and fatty acids, as carbon sources for growth under different nutrient conditions^3,20^. During host infection, pathogenic bacteria are known to up-regulate the glyoxylate cycle^3^.

The glyoxylate bypass is a potential drug target, which could be inhibited by natural or synthetic compounds, such as ITA^21^. ITA strongly inhibits the glyoxylate shunt by acting as a potent competitive inhibitor of ICL^1,2,22^. Inhibition of bacterial growth by ITA exposure has been demonstrated in several bacteria, such as *Pseudomonas indigofera*^1,2^, *Yersinia pestis*^3^, *M*. *tuberculosis* and *S*.*enterica*^4^. For example, Michelucci, *et al*.^4^ reported that supplementation of 25–50 nmol L^−1^ ITA significantly inhibited the growth of *M*. *tuberculosis* and *S*. *enterica*. In our study, the growth of *Vibrio* sp. was completely inhibited in culture media supplemented with 6 mM ITA and reduced growth in the 3 mM ITA treatment compared to the control. Furthermore, glyoxylic acid and other intermediates of the glyoxylate cycle (cis-aconitic acid, isocitric acid and fumaric acid) were significantly higher in the ITA treatment compared to the control. However, most surprisingly, the activity of ICL was not inhibited by ITA in our experiment, which indicates that a different toxic mechanism may be responsible for the antimicrobial effect.

In addition, we observed elevated levels of many amino acids (e.g., alanine, glutamic acid, glycine) in ITA-exposed *Vibrio* sp. Increases in many amino acids have previously been reported in bacteria exposed to environmental stress^23–25^. For example, glutamic acid, which is involved in many metabolic pathways was reported to be synthesized by *Escherichia coli*^26^ and *Vibrio costicola*^27^ to balance the K^+^ uptake from the media. The up-regulation of glutamic acid with increasing temperature was observed in *Vibrio coralliilyticus*^24^. Accumulation of alanine is a common phenomenon in response to various stresses in both plants and animals^28^. Therefore, the level of alanine has been proposed to be a universal stress signal^28^. The increases in alanine were demonstrated in *V*. *parahaemolyticus* exposed to various concentrations of ferric iron^23^. Hence, the accumulation of many amino acids and an activated amino acid metabolism (indicated *via* the pathway analysis) in ITA exposed *Vibrio* sp. may indicate stress responses and disturbance of amino acid metabolism of ITA on *Vibrio* sp.

We observed the accumulation of fatty acids, including pimelic acid, stearic acid, capric acid, and a secondary pathway analysis revealed biosynthesis of unsaturated fatty acids as an altered pathway due to the ITA effect. This may suggest the change of lipid metabolism of *Vibrio* sp. exposed to ITA. However, the mechanisms underlying the increases in these fatty acids is currently unknown. In addition, the accumulation of other organic acids (e.g., lactic acid, adipic acid, malonic acid, maleic acid) remains uncertain.

Among the altered metabolites, trans-4-hydroxyproline (Hyp) was the only down-regulated metabolite in the metabolite profiles of ITA exposed *Vibrio* sp. Hyp is a non-essential amino acid that is a major component of collagen in animals^29^ and glycoproteins in plant cell walls^30^. Hyp is synthesized in plants and animals by hydroxylation of proline by prolyl hydroxylase following protein synthesis (as a post-translational modification). Bacteria are able to metabolize Hyp released by protein degradation of animals and plants^31^. The accumulation of Hyp has been reported in *Thermococcus* spp^32^, *Brevibacterium* sp.^33^ and *Halobacillus halophilus*^25^ under high salt conditions, suggesting that Hyp may be a widespread osmoprotectant in halophilic and halotolerant bacteria. However, we observed the decrease of Hyp in ITA exposed *Vibrio* sp. This may be due to species-specific responses of different bacteria or stressor-specific responses. Nevertheless, these results suggest the important role of Hyp in stress responses of *Vibrio* bacteria, which should be investigated in future studies.

Overall, the findings from this study strongly indicate antimicrobial activity of ITA to marine bacteria. This points toward the potential use of ITA as an antimicrobial metabolite for bacterial control in aquaculture. However, ITA is known to be unsuitable as drug for mammal due to its toxicity to host cells^21^. For marine bivalves, we recently identified the increase of ITA in their tissues during the pathogen exposure^8,9,11^ which indicates ITA is internal metabolite and may not toxic for host at low concentration. However, whether exposure of aquatic organisms to high concentrations of ITA (e.g., 6 mM ITA like in this study) to fight intracellular and drug-resistant bacteria are safe for the host which needs to be investigated prior to application in aquaculture.

## Conclusions

To our knowledge, this is the first study to report antibacterial properties of ITA to a marine *Vibrio* sp. bacterium. After ITA exposure, we observed reduced microbial growth, and higher levels of metabolites in the TCA cycle (glyoxylic acid, cis-aconitic acid, isocitric acid and fumaric acid), amino acids and fatty acids in ITA-exposed bacterial cultures. This indicates that ITA inhibits *Vibrio* sp. growth and disruption of central carbon metabolism and other metabolic changes. However, ICL activity was higher in the ITA treatment compared to the control, suggesting that ITA did not inhibit ICL in the glyoxylate shunt of this *Vibrio* isolate and another toxic mechanism may be responsible for the antimicrobial effect of ITA. Hence, there is a need for future investigations to explore the antimicrobial mechanism of ITA in marine *Vibrio* bacteria and other marine pathogens which may lead to the use of ITA as an antimicrobial compound in aquaculture practices.

## Materials and Methods

### Chemicals

Most of the chemicals used in this study were of analytical grade and obtained from Sigma-Aldrich (St Louis, MO, USA) with the following exceptions: LB broth (Miller, Code: 244610) and thiosulfate citrate bile salts sucrose (TCBS) agar from Fort Richard Laboratories (Auckland, New Zealand), chloroform from Merck (Darmstadt, Germany).

### Bacterial culture and itaconic acid effects

*Vibrio* sp. DO1 (99.5% 16 S sequence similarity with *V*. *corallyliticus* and *V*. *neptunius*; Genbank: EU358784) isolated from Greenshell™ mussel larvae^13^ was kindly provided by Cawthron Institute (Nelson, New Zealand). The bacterial suspension was prepared as previously described^8^ with modifications. Briefly, the bacterial isolates, which were stored at −80 °C in 25% glycerol, were revived by thawing for 1 h prior to incubation in 10 mL volumes of sterilized LB broth at 25 °C for 12 h. The LB broth was prepared by adding 20 g of LB broth powder in 1 L of 5 μm-filtered artificial seawater (FAS), which was then autoclaved at 121 °C for 15 min. The bacterial suspension was streaked on TCBS agar plates and sub-cultured three times to ensure purity. Bacterial colonies were cultured in 20 mL LB for 24 h prior to using for the ITA study.

The effects of ITA on *Vibrio* were assessed by adding 100 μL of *Vibrio* sp. stock (8.7 × 10^7^ cells mL^−1^) into glass bottles containing 100 mL LB with three different ITA concentrations (0, 3, 6 mM) supplemented with 3 mM sodium acetate. The bacterial cultures were incubated at 25 °C and sampled every 6 h up to 24 hpi for bacterial growth using a spectrophotometer (Ultrospec 2100 pro UV–Vis: Biochrom Ltd., Cambridge, UK) to measure absorbance at 600 nm. For metabolomics analyses, bacteria were harvested at 24 hpi. 1 mL of bacterial culture was centrifuged at 2438 × g for 10 minutes at 4 °C on an Eppendorf Centrifuge 5810 R (Eppendorf AG, Hamburg, Germany). The pelleted cells were washed with FAS and re-suspended with 1 mL of FAS. The final cell pellets were flash frozen in liquid nitrogen and stored at −80 °C until metabolite extraction and enzyme analyses could be performed. For isocitrate and isocitrate lyase assays, 1 mL bacterial culture was sampled and stored at −80 °C.

### Isocitrate assay

The concentrations of isocitrate in *Vibrio* sp. samples were measured by isocitrate assay kit (Sigma-Aldrich, St Louis, MO, USA). The assay was performed according to the manufacturer’s protocol. In summary, isocitrate standards containing 0, 4, 8, 12, 16, 20 nmole per well were prepared in a 96-well plate from 20 μL of 100 mM isocitrate stock. One hundred μL of *Vibrio* sp. in LB broth was homogenized in 100 μL of isocitrate assay buffer and centrifuged at 13,000 × g for 10 minutes (Centrifuge 5810 R, Eppendorf AG, Hamburg, Germany) to remove insoluble material. Twenty-five μL of each sample were added into each well and mixed with 25 μL of isocitrate assay buffer. A standard mix containing 46 μL isocitrate assay buffer, 2 μL substrate mix and 2 μL substrate mix was added into each of the wells and mixed. Blank samples contained 48 μL isocitrate assay buffer and 2 μL substrate mix only. All samples, blanks and standards were incubated for 30 min in the dark at room temperature prior to measuring at 450 nm with a microplate reader (Multiskan FC, Thermo, Waltham, MA, USA). Concentrations of isocitrate in the samples were calculated based on absorbance and standard curves.

### Isocitrate lyase assay

Quantification of isocitrate lyase in *Vibrio* sp. samples was conducted following a published protocol^34^. In brief, reagents were prepared and added into suitable cuvettes as follows: 0.50 mL of 50 mM imidazole buffer (pH 6.8 at 30 °C), 0.1 mL of 50 mM magnesium chloride solution (MgCl_2_), 0.1 mL of 10 mM ethylenediaminetetraacetic acid solution (EDTA), 0.1 mL of 40 mM phenylhydrazine hydrochloride solution and 0.1 mL of 10 mM DL-isocitric acid solution (isocitrate). Cuvettes containing the reagent mixture was equilibrated to 30 °C in a water bath. For each *Vibrio* sp. sample, 0.1 mL was combined with the reagent mix immediately measured in a spectrophotometer at 324 nm (Ultrospec 2100 pro UV–Vis: Biochrom Ltd., Cambridge, UK). Samples were placed back in the 30 °C water bath re-measured after 5 min. Alternatively, 0.1 mL of imidazole buffer was added to blanks and similarly measured. ICL concentrations in each sample was calculated based on A_324_ min^−1^ of samples and blanks.

### Metabolite extractions and GC-MS measurements

Metabolites in *Vibrio* sp. samples were extracted in cold methanol-water solution (MeOH:H_2_O, 50% and 80%, sequentially), and derivatized via methyl chloroformate alkylation (MCF), as previously described^35,36^. Derivatized extracts were transferred into 2 mL amber GC glass vials fitted with inserts (Sigma-Aldrich, St. Louis, MO, USA) for GC-MS analyses which were performed on a gas chromatograph GC7890B coupled to a quadrupole mass spectrometer MSD5977A (Agilent Technologies, USA), with a quadrupole mass selective detector (EI) operated at 70 eV. The system was equipped with a ZB-1701 GC capillary column (30 m × 250 μm id × 0.15 μm with 5 m stationary phase) (Phenomenex, Torrance, CA, USA). Helium was used as the carrier gas and was held constant at the flow of 1 mL min^−1^. The instrumental setup parameters for MCF derivatized samples were conducted according to Smart, *et al*.^35^. The injection volume was 1 μL and all samples were injected randomly.

Confirmation of specific metabolites in the TCA cycle/glyoxylate shunt (citric acid, cis-aconitic acid, isocitric acid, succinic acid, fumaric acid, malic acid, glyoxylic acid and pyruvic acid) was conducted by extraction, derivatization and GC-MS measurements of the standards of these compounds (20 μL of 20 mM solutions).

### Quality control for GC-MS measurements

Several types of quality control (QC) samples were employed to ensure reproducibility of GC-MS measurements as previously described^11^. Firstly, 3 blank samples containing 20 μL 10 mM d_4_ alanine were extracted using the sample protocol. Secondly, three standard amino acid mixtures (20 µL, 20 mM) were similarly derivatized with extracted samples. The third type of QC was a pool of all samples. These QC samples were injected at the beginning and after every six samples. In addition, non-derivatized standard alkane mixtures and chloroform solvent were injected at the beginning and at the end of the measurements for system checking. Together, QC samples made up more than 30% of the total injections.

### GC-MS data processing

Raw spectra were processed using Automated Mass Spectral Deconvolution and Identification System (AMDIS) software (version 2.66) integrated with the MassOmics R-based package (The University of Auckland). This process includes baseline correction, peak detection, chromatogram deconvolution and alignment. The target hits were identified using an in-house library (The University of Auckland) with the minimum matching percentage of 70%. Other parameters used to accomplish this analysis were: retention time (RT) window (0.2 min), RT range (6.5–34.0 min), component width (14), m/z range (83–207), and scan sets (3). Annotated metabolites were manually checked with ChemStation software (Agilent Technologies, Inc., US) and AMDIS for the presence of contaminants. Repeats (based on ID number, match factor and retention time) and aberrant records were removed. Data were normalized by the internal standard (d_4_ alanine) and absorbance of the bacterial cultures to compensate for potential technical variations (e.g. variable metabolite recoveries) and differences in biomass amongst the treatments, respectively.

### Statistical and pathway analysis

Metabolite profile data were analysed using MetaboAnalyst 4.0^37^. Data were normalized by generalized logarithm (glog) transforming and mean centring to make individual features more comparable. Multivariate analyses, including unsupervised PCA and supervised PLS-DA were used to assess variability among samples and between sample classes. Validation of the PLS-DA model was performed using leave one out cross validation (LOOCV), which was assessed via accuracy, R^2^ and Q^2^ values^38^. Univariate analysis was performed using *t*-test to identify differences between metabolite profiles of ITA-treated and control cultures of *Vibrio* sp. A heatmap of altered metabolites was generated to assess the abundance of these metabolites (low/high) via intuitive visualization. Classical univariate ROC analyses for individually altered metabolites in glyoxylate shunt and multivariate ROC analysis (using linear support vector machines) for all of these features were performed to assess the accuracy of biomarker models.

Quantitative enrichment analysis (QEA) using global test algorithm^39^ and network topology analysis (NTA) using relative-betweeness centrality^40^ were performed to investigate functional relationships among the annotated metabolites using the *Escherichia coli* K-12 MG1655 reference pathway library in the Kyoto Encyclopedia of Genes and Genomes (KEGG) database. Pathways involving two or more annotated metabolites with simultaneous QEA p-values < 0.05, QEA false discovery rates (FDRs) < 0.1, and with NTA pathway impact (PI) scores > 0.1 were considered as primary pathways of interest due to ITA effects.

## Supplementary information

Supplementary Table 1
