# Supplementary material for: Itaconic acid inhibits growth of a pathogenic marine Vibrio strain: A metabolomics approach
Source: Sci Rep. 2019 Apr 11;9:5937. doi: 10.1038/s41598-019-42315-6 (PMC6459830; doi:10.1038/s41598-019-42315-6)
Supplement: Supplementary file 1 — Supplementary Table 1 [file 41598_2019_42315_MOESM1_ESM.docx]

**Itaconic acid inhibits growth of a pathogenic marine *Vibrio* strain: A metabolomics approach**

Thao V Nguyen, Andrea C. Alfaro, Tim Young, Saras Green, Erica Zarate, and Fabrice Merien

**Supplementary table 1**. List of metabolites identified as significantly different between the ITA treatment and the control by *t*-test (p < 0.05).

| **Compounds** | **t.stat** | **p.value** | **FDR** |
| --- | --- | --- | --- |
| Benzoic acid | -9.683 | <0.001 | <0.001 |
| cis-Aconitic acid | -7.669 | <0.001 | <0.001 |
| NADP_NADPH | -5.641 | <0.001 | 0.003 |
| Adipic acid | -5.533 | <0.001 | 0.003 |
| trans-4-Hydroxyproline | 4.511 | 0.001 | 0.010 |
| Malonic acid | -4.035 | 0.002 | 0.019 |
| Fumaric acid | -3.838 | 0.003 | 0.019 |
| Maleic acid | -3.838 | 0.003 | 0.019 |
| Capric acid | -3.828 | 0.003 | 0.019 |
| Glyoxylic acid | -3.696 | 0.004 | 0.021 |
| 2-Oxoglutaric acid | -3.655 | 0.004 | 0.021 |
| Alanine | -3.358 | 0.007 | 0.033 |
| 4-Aminobutyric acid (GABA) | -3.088 | 0.011 | 0.042 |
| Stearic acid | -3.083 | 0.012 | 0.042 |
| Leucine | -3.069 | 0.012 | 0.042 |
| Hexanoic acid | -3.059 | 0.012 | 0.042 |
| 2-Hydroxyglutaramic acid | -2.938 | 0.015 | 0.046 |
| Lactic acid | -2.919 | 0.015 | 0.046 |
| Pimelic acid | -2.916 | 0.015 | 0.046 |
| Histidine | -2.852 | 0.017 | 0.048 |
| Glycine | -2.794 | 0.019 | 0.048 |
| Isoleucine | -2.787 | 0.019 | 0.048 |
| Glutamic acid | -2.785 | 0.019 | 0.048 |
| Phenylalanine | -2.763 | 0.020 | 0.048 |
